# Simultaneous determination of eight analytes of Fuzheng Huayu recipe in beagle dog plasma by UHPLC–Q/exactive Orbitrap HRMS and its application to toxicokinetics

**DOI:** 10.1002/bmc.5329

**Published:** 2022-01-22

**Authors:** Yu‐Lin Wang, Ming‐jv Yang, Rong‐sheng Li, Ye‐qing Hu, Yi‐feng Pan, Tao Yang

**Affiliations:** ^1^ Department of Cardiology, Institute of Cardiovascular Disease of Integrated Traditional Chinese Medicine and Western Medicine Shuguang Hospital affiliated to Shanghai University of Traditional Chinese Medicine Shanghai China; ^2^ Shanghai Key Laboratory of Traditional Chinese Clinical Medicine Shanghai China; ^3^ Grade 2018 Tianjin University of Traditional Chinese Medicine Tianjin China; ^4^ Shanghai Modern Chinese Medicine Co. Ltd China

**Keywords:** beagle dog, Fuzheng Huayu recipe, toxicokinetics, traditional Chinese medicine, UHPLC–Q‐Exactive Orbitrap HRMS

## Abstract

Fuzheng Huayu recipe (FZHY) is a Chinese patent medicine for the treatment of liver fibrosis. This study aimed to investigate the toxicokinetics of FZHY in beagle dogs after oral administration. Blood samples were collected on days 1, 15 and 28 after oral gavage of FZHY dosages of 400 or 1,200 mg/kg body weight once a day. A UHPLC–Q‐Orbitrap method was developed and validated to simultaneously determine and quantify eight components of FZHY in beagle dog plasma. The times to peak concentration for eight components were18–120 min. The peak concentrations (*C*_max_) of amygdalin, genistein, daidzein and 3,4‐dihydroxybenzaldehyde were 1.43–43.50 ng/ml, the areas under the concentration–time curve (AUC_(0–*t*)_) were 2.45–6,098.25 ng min/ml, and the apparent volumes of distribution (*V*_d_) were 0.05–131.23 × 10^4^ ml/kg. The values of *C*_max_ of prunasin, schisantherin A, schisandrin A and schisandrin were 7.35–1,450.73 ng/ml, the values of AUC_(0–*t*)_ were 3,642.30–330,388.65 ng min/ml, and the values of *V*_d_ were 11.15–1,087.18 × 10^4^ ml/kg. No obvious accumulation of the eight compounds was observed in beagle dogs. The results showed that the method is rapid, accurate and sensitive, and is suitable for detecting the eight analytes of FZHY. This study provides an important basis for the assessment of FZHY safety.

## 1 INTRODUCTION

Fuzheng Huayu (FZHY) recipe comprises six traditional Chinese medicinal herbs, namely, Radix Salvia Miltiorrhizae (Danshen), Cordyceps (Chongcao), Semen Persicae (Taoren), Gynostemma Pentaphyllammak (Jiaogulan), Pollen Pini (Songhuafen) and Fructus Schisandrae Chinensis (Wuweizi), and has effects including strengthening vital energy, promoting blood circulation to prevent blood stasis, invigorating the kidneys and nourishing the liver (Wang et al., 2015). FZHY was developed by Shanghai University of Traditional Chinese Medicine according to traditional Chinese medicine (TCM) theory. FZHY Tablet is a proprietary Chinese medicine made by Shanghai Modern Chinese Medicine Co. Ltd, that is based on the FZHY recipe and shows outstanding effects in the prevention and treatment of chronic liver disease, such as improving patient symptoms, alleviating disease and preventing complications (Hai et al., 2009). In China, FZHY has been confirmed to be effective for treating liver fibrosis in tens of thousands of clinical cases (Guan et al., 2021). FZHY Tablet has been listed as a first‐line anti‐fibrotic drug in the *Guidelines for the Prevention and Treatment of Liver Fibrosis with Integrated Traditional Chinese and Western Medicine* and the *Consensus on Diagnosis and Treatment of Liver Cirrhosis with Integrated Traditional Chinese and Western Medicine* (Xu & Liu, 2019). Furthermore, FZHY Tablet was approved by the US Food and Drug Administration (FDA) in 2006 and directly entered phase II clinical trials without phase I trials. This multicenter phase II clinical trial also showed that FZHY Tablet has good efficacy against anti‐hepatic fibrosis (Hassanein et al., 2014). FZHY Tablet has become the first Chinese patent medicine in the field of liver disease to undergo phase III clinical research in the USA.

Although FZHY has long been used clinically, research has continued owing to its complex components. FZHY in mice has been reported to prevent the development of liver fibrosis (Dong et al., 2018; Jiang et al., 2020; Yang et al., 2020). Presently, research on FZHY is mainly focused on its antiliver fibrosis mechanism (Chen et al., 2019; Hu et al., 2019; Wu et al., 2019; Zhang et al., 2020) and material basis. A total of 49 blood components of FZHY have been identified by ultra‐performance liquid chromatography–high resolution time‐of‐flight mass spectrometry (UHPLC–Q‐TOF/MS) (Wu et al., 2019). In our previous studies, 11 compounds and two metabolites of Danshen in FZHY were identified in rat plasma (Yang et al., 2013), and the pharmacokinetics and tissue distribution of 10 components of FZHY in Wistar rats, including schisandrin B and rosmarinic acid, were studied (Yang, Liu, Wang, et al., 2015a; Yang, Liu, Zheng, et al., 2015). However, the toxicokinetics of FZHY have not been reported.

Toxicokinetics has become an important nonclinical toxicity test (Meng et al., 2014). As an important part of TCM safety evaluation, toxicokinetics research can use toxicological tests to evaluate possible adverse reactions to human drugs, and effectively interpret the results of toxicity tests to predict the safety of medicines in humans (Gao et al., 2015). Beagle dogs are nonrodents that are suitable for evaluating drug safety. However, owing to the high costs and experimental challenges, there are few studies on the toxicokinetics of traditional Chinese medicines in dogs. FZHY itself is characteristic of ‘multicomponent, multistep, multitarget’ intervention, and has unique and complex properties. Therefore, toxicokinetics research must also be conducted on FZHY using beagle dogs to facilitate further scientific research. Drug safety and quantitative evaluation of drug absorption, distribution, metabolism and excretion in the body can fully determine the dose–effect and time–effect relationships between the toxicity and ingredients of FZHY.

Therefore, in this study, eight chemical components in beagle dog plasma were determined by liquid chromatography–tandem mass spectrometry, and a toxicokinetic study of FZHY tablets after oral administration in beagle dogs was conducted. This study aimed to promote phase III clinical trials in the USA, explore the toxicokinetic characteristics of FZHY, provide toxicokinetic data support for subsequent experimental research and further scientific medication guidance, index monitoring and risk control, and also improve rational guidance for clinical drug use.

## 2 MATERIALS AND METHODS

### 2.1 Materials and reagents

Osalmid was purchased from Sigma‐Aldrich Co. (St Louis, MO, USA) for use as the internal standard (IS). Schisandrin (7432‐28‐2), schisandrin A (61281‐38‐7), schisantherin A (58546‐56‐8), amygdalin (29883‐15‐6), genistein (446‐72‐0), 3,4‐dihydroxybenzaldehyde (139‐85‐5) and daidzein (111502‐200402) were purchased from the China Institute for Food and Drug Control (Beijing, China). Prunasin (99‐18‐3) was purchased from Shanghai Standard Technical Service Co. Ltd (Shanghai, China).

The purity of all reference standards was ≥99.5%. Ultrapure water was obtained from a Milli‐Q Academic System (Millipore, Billerica, MA, USA). HPLC‐grade acetonitrile and methanol were purchased from Fisher Scientific Co. (Fair Lawn, NJ, USA). HPLC‐grade formic acid was obtained from CNW Technologies GmbH (Düsseldorf, Germany). All other chemicals were analytical reagents.

FZHY recipe tablets (lot no. S190901) were prepared and provided by Shanghai Huanghai Pharmaceutical Co. Ltd (Shanghai, China). According to the human dosage in clinical practice (80 mg/kg/day) and the human–dog coefficient of skin surface area, FZHY tablets were suspended in distilled water and administered orally at doses of 400 and 1,200 mg/kg (human equivalent dose).

### 2.2 Animals and ethics statement

Eight healthy male and female beagle dogs, aged 6–9 months, were obtained from Beijing Max Biotechnology Co. Ltd (Beijing, China). Appropriate animals were selected according to their health status and weight during the adaptation period. According to the weight balance principle, the group‐based method in Provantis v9.4.3.0 (PV‐02) was used for random grouping (SOP: CSV‐043‐04). Male and female animals were randomly assigned to avoid assignment to the same group of the same sex in the same litter, with the weight difference between male and female animals not exceeding 20% of the average weight. The animal room temperature was controlled at 18–26°C, and the relative humidity was within the range of 40–70%. The light–dark cycle was controlled for 12 h, and the animals received natural light every day. This study followed the relevant policies and guiding principles of animal welfare of the Shanghai Institute of Pharmacy, Chinese Academy of Sciences, and was approved by the Laboratory Animal Use and Management Committee (no. 2019‐08‐RJ‐195).

### 2.3 Liquid chromatography–mass spectrometry conditions

A Thermo Scientific UHPLC–Q‐Orbitrap system equipped with a heated electrospray ionization source and a Dionex Ultimate 3000 system (Thermo Fisher Scientific, USA) was used. Chromatographic separation was conducted using an Acquity HSS T3 column (2.1 × 100 mm, 1.8 μm) maintained at 45°C. The mobile phase consisted of 0.1% formic acid–water (C) and acetonitrile (D). The gradient elution conditions were as follows: 0–12 min, 5% D; 12–14 min, 95% D; 14.1–16 min, 5% D. The flow rate was kept at 0.3 ml/min and the injection volume was 3 μl.

Mass spectrometry was conducted in positive and negative modes using the following operating parameters: sheath gas pressure, 35 arb; auxiliary gas pressure, 10 arb; capillary temperature, 320°C; heater temperature, 300°C; scan mode, full MS (resolution 70,000); scan range, *m*/*z* 150–1,200.

### 2.4 Preparation of stock solutions, working standard solutions and quality control samples

#### 2.4.1 Preparation of stock and working standard solutions

**FIGURE 1:**
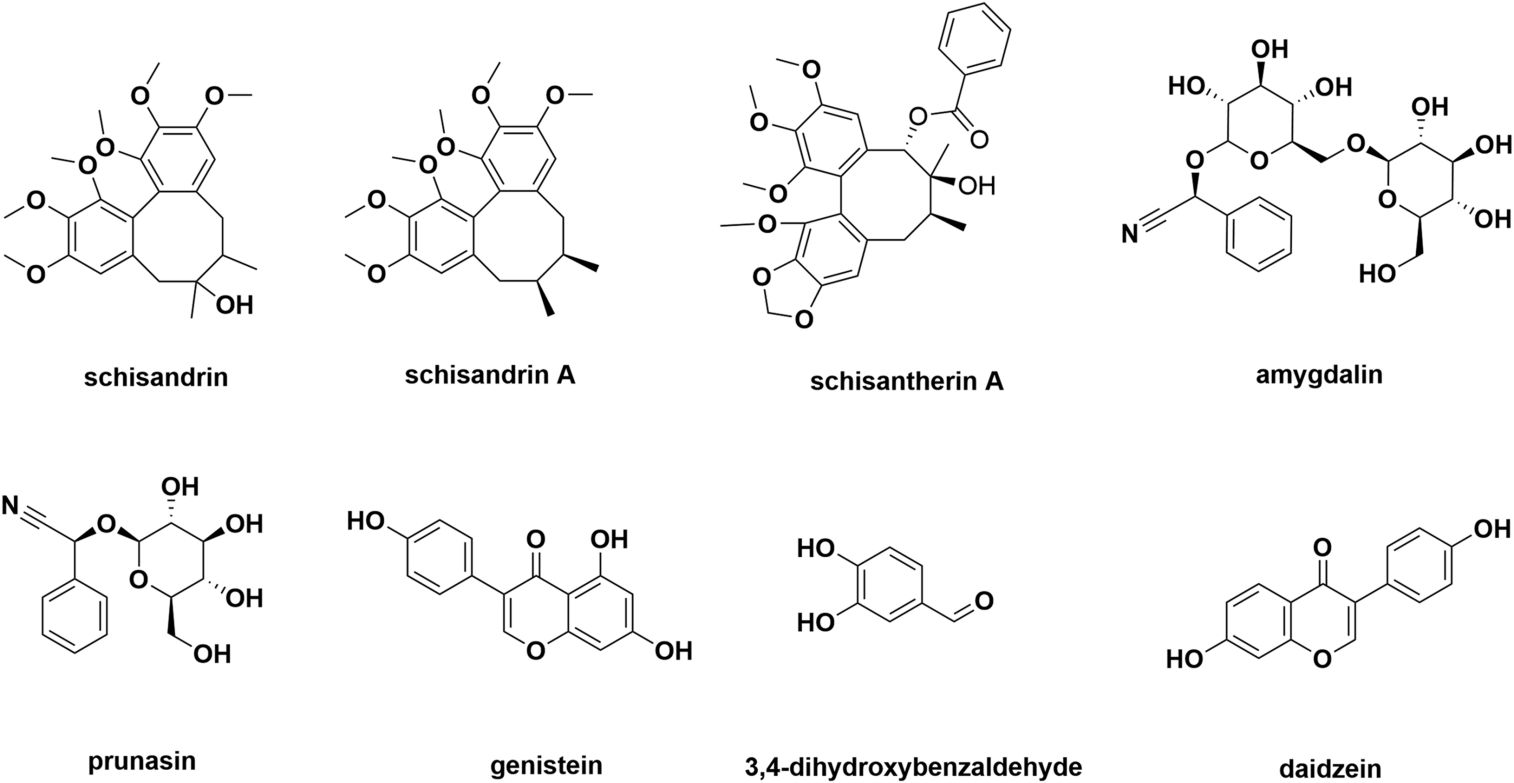
Chemical structures of schisandrin, schisandrin A, schisantherin A, amygdalin, prunasin, genistein, 3,4‐dihydroxybenzaldehyde and daidzein

Stock solutions of eight analytes (schisandrin, schisandrin A, schisantherin A, amygdalin, prunasin, genistein, 3,4‐dihydroxybenzaldehyde and daidzein) were prepared in acetonitrile at a concentration of 500 μg/ml and stored at −20°C. (structers are shown in Figure 1). Each analyte was weighed for the calibration standards and three quality control (QC) samples. Working solutions were prepared by mixing all eight analytes, resulting in a concentration of 5 mg/ml for each analyte. The mixture was serially diluted with acetonitrile to obtain calibrated working solutions ranging from 1,066.667 to 0.013 ng/ml. Osalmid (IS) was dissolved in acetonitrile to a concentration of 1 mg/ml, further diluted with acetonitrile to 363.64 ng/ml, and kept at −20°C.

#### 2.4.2 Preparation of quality control samples

Blank plasma samples (50 μl) from six different batches were taken and mixed with working solutions (150 μl) of different concentrations containing IS (266.67 ng/ml). The sample mixture was vortex‐mixed for approximately 1 min and centrifuged at 12,000 rpm for 10 min at 4°C. The supernatant was then transferred to an Eppendorf tube and the required QC samples with low (LQC), medium (MQC), and high (HQC) concentrations were prepared separately. All solutions were stored at −20°C.

### 2.5 Preparation of plasma sample

Plasma samples were taken out of the refrigerator (−80°C) and allowed to thaw naturally at room temperature. Plasma samples (50 μl) were taken into a 1.5 ml Eppendorf tube and mixed with acetonitrile (150 μl) containing IS working solution (266.67 ng/ml) to precipitate the protein. After vortexing for 1 min and centrifuging at 12,000 rpm for 10 min at 4°C, the supernatant was transferred to labeled vials and 5 μl of the solution was immediately injected into the UHPLC–Q‐Orbitrap system for analysis.

### 2.6 Method validation

According to the Bioanalytical Method Validation Guidance for Industry of the FDA, the UHPLC–Q‐Exactive HRMS method for determination of eight components in beagle plasma was evaluated, including the selectivity, linearity, detection limit and quantitative limit, matrix effects, precision, accuracy and stability.

#### 2.6.1 Selectivity

The selectivity was evaluated by comparing blank plasma from six batches of blank beagle dog plasma, with blank plasma samples spiked with IS (266.67 ng/ml), and working standard solutions at lower limit of quantitation (LLOQ) level and beagle dog plasma sample after oral administration of FZHY at a dose of 400 mg/kg at 360 min to evaluate the interferences of endogenous compounds. No interference of the target chemical components was observed from endogenous substances.

#### 2.6.2 Calibration curve, lower limit of quantification and limit of detection

A series of standard working solutions with concentrations of 800, 320, 128, 51.20, 20.48, 8.19, 3.28, 1.32, 0.53, 0.21, 0.08, 0.03 and 0.01 ng/ml were prepared according to the working solution sample preparation method described in Section 2.4.1, and the IS solution concentration was 200 ng/ml, as detected by a UHPLC–Q‐Orbitrap from low to high concentration. The linearity of the calibration curve containing eight nonzero concentrations was determined by plotting the peak area ratios of the target compound to IS (analyte/IS, *y*) against the different concentrations of mixed working standard solutions using a weighted least squares linear regression model (1/*x* ^2^). Simultaneously, the linearity and correlation coefficient of the target components were also obtained. The LLOQ was defined as the lowest possible drug concentration reproducibly detected. The signal‐to‐noise ratio at this concentration was at least 10 times higher than that of the blank plasma. The limit of detection (LOD) was considered the lowest component concentration with a signal‐to‐noise signal ratio >3 after diluting to the lowest concentration point in the linear range.

#### 2.6.3 Matrix effects and recovery

Blank plasma samples from six different sources were extracted and then spiked with the analytes into the low, medium and high QCs with the IS. Matrix effects were determined by comparing the analyte peak area in the plasma samples with the peak area in water–acetonitrile mixtures. The recovery was determined by comparing the peak response of pre‐extracted blank plasma samples with post‐extracted plasma samples. Both the recovery and matrix effects should be consistent with 85–115%, and the LQC should be consistent with 85–120%.

#### 2.6.4 Accuracy and precision

Within‐run and between‐run accuracy and precision were assessed by replicate analyses of QCs at four concentration levels (LLOQ and low, medium and high QC samples), with six replicates each. To evaluate the within‐run accuracy and precision, the same sample was validated three times within 24 h, while the between‐run accuracy and precision were determined by assaying the same samples on three consecutive days. The coefficient of variation (CV), used to measure precision, was calculated as follows: CV (%) = [*σ*/*μ*/*μ*] × 100, where *σ* and *μ* represent the standard deviation and average value of peak area ratio, respectively. The accuracy was determined using the ratio of the calculated concentration to the nominal concentration at each QC level (calculated value/nominal value × 100).

#### 2.6.5 Stability

The stability was evaluated by analyzing six replicates at two QC levels (low and high concentrations) under different conditions (autosampler, benchtop, freeze–thaw, and long‐term stability). Briefly, the autosampler stability was determined by reinjecting the reconstituted QCs after 24 h storage at 10°C. The benchtop stability was evaluated in low‐ and high‐concentration QC samples after storage for 10 h at room temperature (25 ± 2°C). The freeze–thaw stability was determined using QC samples after three cycles of freezing at −80°C and thawing at room temperature, and the long‐term stability was determined by analyzing QC samples after storage at −80°C for 30 days. The accuracy (nominal percentage; mean ± SD, ng/ml) at each level should be ±15% for acceptance criteria. The acceptable precision (CV, %) should be within ±15% and the low‐concentration QC samples should not exceed 20%.

### 2.7 Toxicokinetics study

#### 2.7.1 Animals, drug administration, and sampling

To explore initial tolerability and toxicokinetics, an acute oral toxicity study was conducted in dogs using the maximum tolerated dose method. Healthy male and female dogs were randomly assigned to two groups (four dogs per group). Dogs received FZHY by oral gavage at dosages of 400 (low‐dose group) and 1,200 mg/kg body weight (high‐dose group) once a day for 4 weeks. Blood samples (250 μl) were collected from the right forelimb vein on days 1, 15 and 28 at 0, 15, 30, 60, 120, 360 and 1,440 min after dosing. The blood samples were centrifuged at 3,500 rpm for 10 min and the supernatant was stored in an Eppendorf tube at −80°C.

#### 2.7.2 Data analysis

Xcalibur 4.1 software was used to analyze and process the data, and the ratio of each component to the IS peak area was substituted into the standard curve to calculate the blood concentrations of dogs with different dosages at different time points. The main toxicokinetic parameters were analyzed using PK solution 2 software (Summit Research Service, USA), including the maximal plasma concentration (*C*_max_), time to reach *C*_max_ (*T*_max_), area under the plasma concentration–time curve (AUC_(0*–t*)_ and AUC_(0–*∞*)_), and apparent volume of distribution (*V*_d_). SPSS V26.0 software was used for data analysis. The data were subjected to the normal distribution, expressed by the mean and standard deviation (mean ± SD), otherwise expressed by the median and interquartile spacing (*M*[P25, P75]). Two different groups with normally distributed data were compared using Student’s *t*‐test and non‐normally distributed data were analyzed using the Kruskal–Wallis test. A value of *P* < 0.05 was considered statistically significant.

## 3 RESULTS AND DISCUSSION

### 3.1 Method validation

#### 3.1.1 Selectivity

**FIGURE 2:**
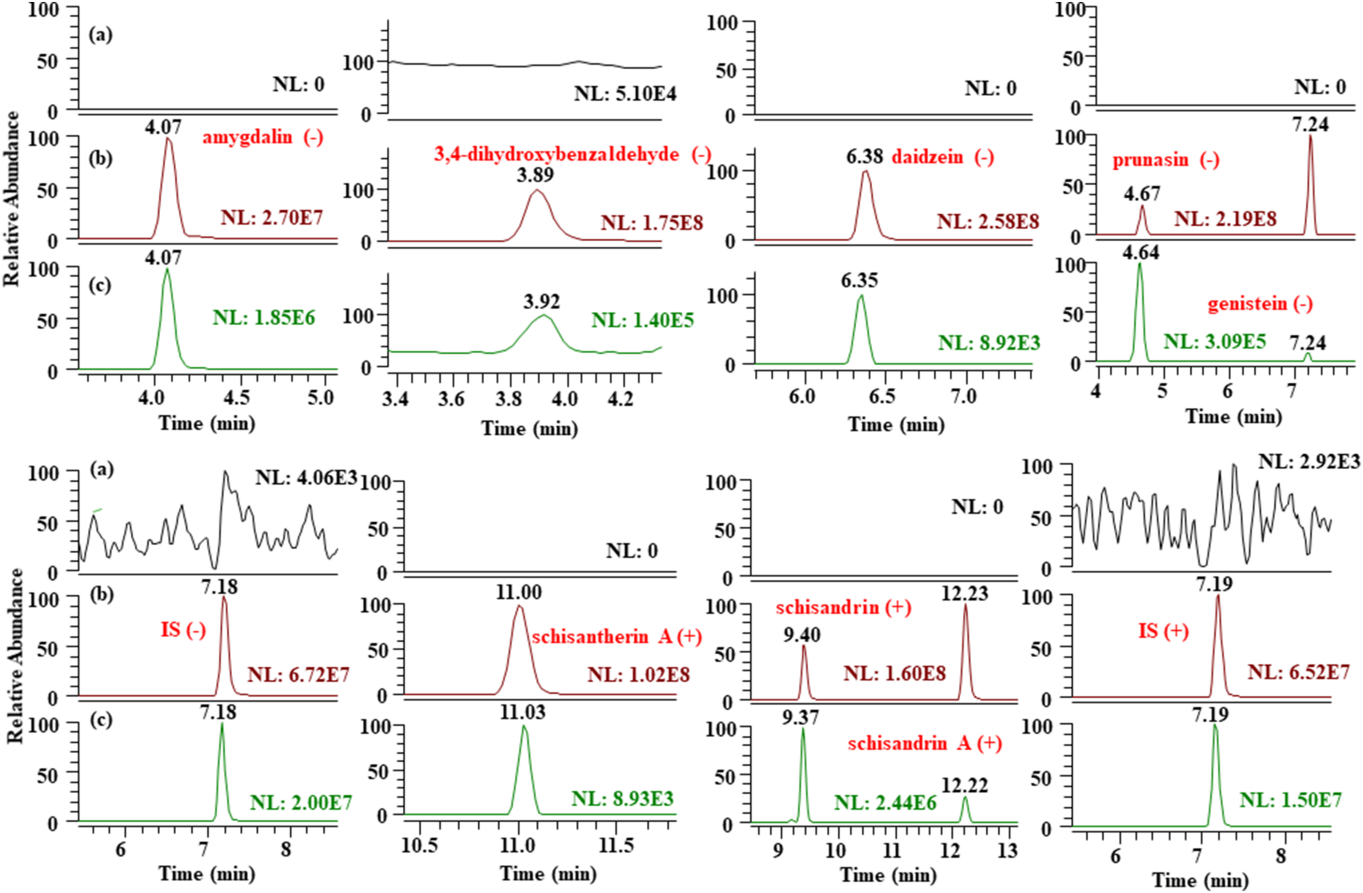
Total ion chromatogram and representative chromatograms of the eight components in beagle dogs. (a) Blank plasma sample. (b) Blank plasma samples spiked with schisandrin (9.40 min), schisandrin A (12.23 min), schisantherin A (11.00 min), amygdalin (4.07 min), prunasin (4.67 min), genistein (7.24 min), 3,4‐dihydroxybenzaldehyde (3.89 min), daidzein (6.38 min), internal standard (IS) (7.19/7.18 min) 8 standards at LLOQ level and the IS at 266.67 ng/ml. (c) Beagle dog plasma sample after oral administration of FZHY at a dose of 400 mg/kg at 360 min

Chromatograms of the LLOQ samples, and mixtures comprising six batches of blank beagle dog plasma with the corresponding spiked plasma containing IS and the beagle dog plasma sample after oral administration of FZHY, are shown in Figure 2. There was no interference of the target chemical components by endogenous substances.

#### 3.1.2 Calibration curve, lower limit of quantification and limit of detection

A weighted (1/*x* ^2^) least squares linear regression model was used to assess the linearity achieved over a calibration range of 0.21–2,000 ng/ml in dog plasma. The LLOQ values of schisandrin, schisandrin A, schisantherin A, amygdalin, prunasin, genistein, daidzein and 3,4‐dihydroxybenzaldehyde were 1.32, 0.21, 0.21, 0.08, 0.53, 0.08, 0.08 and 0.03 ng/ml, respectively. The LOD values of schisandrin, schisandrin A, schisantherin A, amygdalin, prunasin, genistein, daidzein and 3,4‐dihydroxybenzaldehyde were 0.21, 0.08, 0.08, 0.03, 0.08, 0.03, 0.03 and 0.01 ng/ml, respectively. The calibration curve, correlation coefficient (*r* ^2^), linear range and more detailed information regarding the eight analytes are shown in Table S1.

#### 3.1.3 Matrix effects and extraction recovery

Protein precipitation resulted in an overall recovery of 85–115% for all eight analytes at low, medium and high QC concentrations, and the recovery was consistent over the investigated concentration range, at 85–115%. Overall, the plasma samples were extracted efficiently by protein precipitation and this method can be extensively used for purification, as indicated by the minor matrix effects observed (Table 1).

**TABLE 1:** Summary of recovery, matrix effects, and within‐ and between‐run accuracy of the eight analytes obtained using the UHPLC–ESI–MS method (*n* = 6)

Analytes	Nominal level	Recovery (%)	Matrix effect (%)	Within‐run		Between‐run	
		Mean ± SD (%)	Mean ± SD (%)	Mean ± SD (%)	CV	Mean ± SD (%)	CV
Schisandrin	LLOQ			90.08 ± 8.02	5.54	89.99 ± 5.62	4.23
	LQC	103.29 ± 14.88	85.17 ± 5.66	89.90 ± 4.33	5.31	92.20 ± 4.35	14.88
	MQC	95.70 ± 6.01	102.93 ± 3.43	95.98 ± 5.30	5.36	91.56 ± 4.23	5.6
	HQC	100.69 ± 10.39	104.83 ± 5.66	102.95 ± 7.28	3.62	101.29 ± 5.21	4.59
Schisandrin A	LLOQ			85.74 ± 5.32	4.12	88.89 ± 8.54	8.9
	LQC	99.52 ± 12.89	98.11 ± 10.74	90.56 ± 5.45	4.04	90.04 ± 4.43	14.62
	MQC	89.74 ± 7.58	92.55 ± 13.21	104.59 ± 7.43	2.95	91.29 ± 5.43	7.67
	HQC	101.52 ± 12.89	101.60 ± 6.08	101.59 ± 8.62	2.61	101.01 ± 9.34	0.69
Schisantherin A	LLOQ			88.49 ± 8.17	3.1	89.45 ± 3.21	10.04
	LQC	106.14 ± 13.42	100.06 ± 13.42	92.90 ± 5.34	4.71	88.74 ± 6.43	9.97
	MQC	101.65 ± 14.10	86.70 ± 14.74	102.45 ± 5.43	3.56	105.56 ± 7.62	2.6
	HQC	103.71 ± 13.42	98.08 ± 11.11	103.90 ± 3.43	3.31	104.36 ± 3.21	3.8
Amygdalin	LLOQ			92.67 ± 6.97	2.98	99.66 ± 4.32	9.79
	LQC	103.36 ± 9.53	107.76 ± 11.23	96.77 ± 6.46	9.04	90.65 ± 3.87	1.12
	MQC	94.38 ± 9.06	102.18 ± 7.41	94.16 ± 4.78	2.95	101.87 ± 4.65	5.18
	HQC	98.73 ± 2.79	94.69 ± 3.45	104.24 ± 5.67	2.66	113.28 ± 8.67	5.74
Prunasin	LLOQ			81.46 ± 7.32	2.99	90.89 ± 5.24	8.34
	LQC	101.92 ± 4.62	100.86 ± 6.54	96.17 ± 3.45	1.34	88.89 ± 7.12	3.83
	MQC	98.27 ± 3.96	98.90 ± 3.28	105.35 ± 9.54	2.84	100.22 ± 5.66	4.88
	HQC	98.82 ± 2.75	98.23 ± 3.20	105.34 ± 5.46	1.58	105.06 ± 5.05	4.83
Genistein	LLOQ			90.01 ± 4.56	3.87	90.78 ± 6.01	7.3
	LQC	102.32 ± 4.85	95.94 ± 5.52	94.60 ± 6.32	2.86	89.90 ± 5.89	3.68
	MQC	98.97 ± 3.20	98.12 ± 1.89	97.24 ± 7.02	2.69	98.54 ± 4.07	4.21
	HQC	99.30 ± 2.49	99.86 ± 0.93	102.53 ± 5.78	1.71	100.78 ± 8.43	4.83
3,4‐Dihydroxybenzaldehyde	LLOQ			91.06 ± 4.44	4.2	88.02 ± 4.03	4.1
	LQC	104.56 ± 7.53	101.07 ± 8.42	91.45 ± 3.45	1.23	92.67 ± 6.35	2.38
	MQC	93.88 ± 2.78	108.57 ± 7.16	98.68 ± 6.43	2.81	102.01 ± 3.87	3.72
	HQC	99.67 ± 2.28	105.75 ± 0.94	103.28 ± 6.84	1.41	105.27 ± 5.72	4.45
Daidzein	LLOQ			100.02 ± 7.43	3.79	89.12 ± 3.94	4.05
	LQC	103.61 ± 5.34	96.15 ± 5.58	92.28 ± 5.43	2.9	91.17 ± 7.61	1.71
	MQC	98.89 ± 5.51	98.29 ± 3.83	98.59 ± 8.45	2.31	96.56 ± 5.18	4.83
	HQC	98.33 ± 1.74	105.75 ± 0.94	101.49 ± 8.54	1.99	110.06 ± 6.34	3.93

CV, Coefficient of variation; LLOQ, lower limit of quantitation; LQC, low quality control; MQC, medium quality control; HQC, high quality control.

#### 3.1.4 Accuracy and precision

The within‐ and between‐run accuracy and precision results for detection of the LLOQ and low, medium and high‐concentration QC samples are presented in Table 1. At each concentration level, the within‐ and between‐run precisions (CV, %) were <15.00%. All samples showed accuracy within the specified limits of 85–115%. The within‐ and between‐run accuracy and precision results of the assay were all within those outlined by the FDA bioanalytical method validation guidance.

#### 3.1.5 Stability

The autosampler, benchtop, freeze–thaw, and long‐term stability of the eight analytes were determined, as shown in Table 2. All stability experiments showed a good measurement precision with CVs of ≤8%. QC samples of two concentration levels (low and high QC) were stable in the autosampler, with a CV of <7.0%, and after storing at room temperature (25 ± 2°C) for 10 h, with a CV of <8%. Samples could undergo three cycles of freezing at −80°C and thawing at room temperature without affecting the stability of the eight analytes, as indicated by a CV of <7%. Furthermore, 30 days of storage at −80°C did not significantly change the concentration of analytes in the plasma.

**TABLE 2:** Stability of the eight analytes (mean ± SD, CV, *n* = 6)

Stability	Nominal levels	Schisandrin	Schisandrin A	Schisantherin A	Amygdalin	Prunasin	Genistein	3,4‐Dihydroxybenzaldehyde	Daidzein
Autosampler (ng/ml, %)	LQC	20.32 ± 0.98	19.89 ± 1.00	19.26 ± 1.00	20.25 ± 0.87	20.65 ± 1.10	19.34 ± 0.81	22.87 ± 0.71	19.46 ± 1.18
		4.81	5.03	5.21	4.32	5.32	4.21	3.12	6.08
	HQC	819.50 ± 46.3	820.60 ± 35.44	802.89 ± 48.98	802.67 ± 33.95	815.04 ± 54.20	819.67 ± 27.21	811.92 ± 26.55	802.19 ± 60.64
		5.65	4.32	6.1	4.23	6.65	3.32	3.27	7.56
Benchtop (ng/ml, %)	LQC	20.39 ± 0.92	19.87 ± 1.02	19.36 ± 1.13	20.07 ± 1.11	19.20 ± 1.16	19.04 ± 0.91	19.52 ± 0.86	19.37 ± 1.40
		4.54	5.11	5.86	5.55	6.02	4.76	4.43	7.23
	HQC	800.39 ± 40.10	798.01 ± 50.43	795.67 ± 32.07	802.89 ± 57.17	806.53 ± 43.07	794.57 ± 59.04	795.38 ± 33.64	800.41 ± 24.89
		5.01	6.32	4.03	7.12	5.34	7.43	4.23	3.11
Freeze–thaw (ng/ml, %)	LQC	21.77 ± 1.21	21.77 ± 1.21	20.22 ± 0.87	19.43 ± 0.59	20.90 ± 1.16	19.82 ± 0.92	20.35 ± 0.65	19.21 ± 0.98
		5.54	5.54	4.32	3.03	5.55	4.63	3.21	5.09
	HQC	799.90 ± 52.15	799.90 ± 52.15	801.56 ± 47.05	796.08 ± 33.67	796.70 ± 34.34	798.21 ± 16.92	794.50 ± 24.55	798.82 ± 41.14
		6.52	6.52	5.87	4.23	4.31	2.12	3.09	5.15
Long‐term stability (ng/ml, %)	LQC	21.89 ± 1.11	19.01 ± 1.43	19.50 ± 0.63	20.40 ± 0.84	19.82 ± 1.24	19.02 ± 1.01	19.56 ± 0.80	19.20 ± 0.99
		5.09	7.54	3.23	4.14	6.26	5.32	4.07	5.15
	HQC	802.01 ± 29.03	798.88 ± 26.68	796.18 ± 34.32	803.23 ± 40.80	797.76 ± 25.69	798.97 ± 51.37	800.00 ± 43.52	799.08 ± 42.59
		3.62	3.34	4.31	5.08	3.22	6.43	5.44	5.33

### 3.2 Toxicokinetics

**FIGURE 3:**
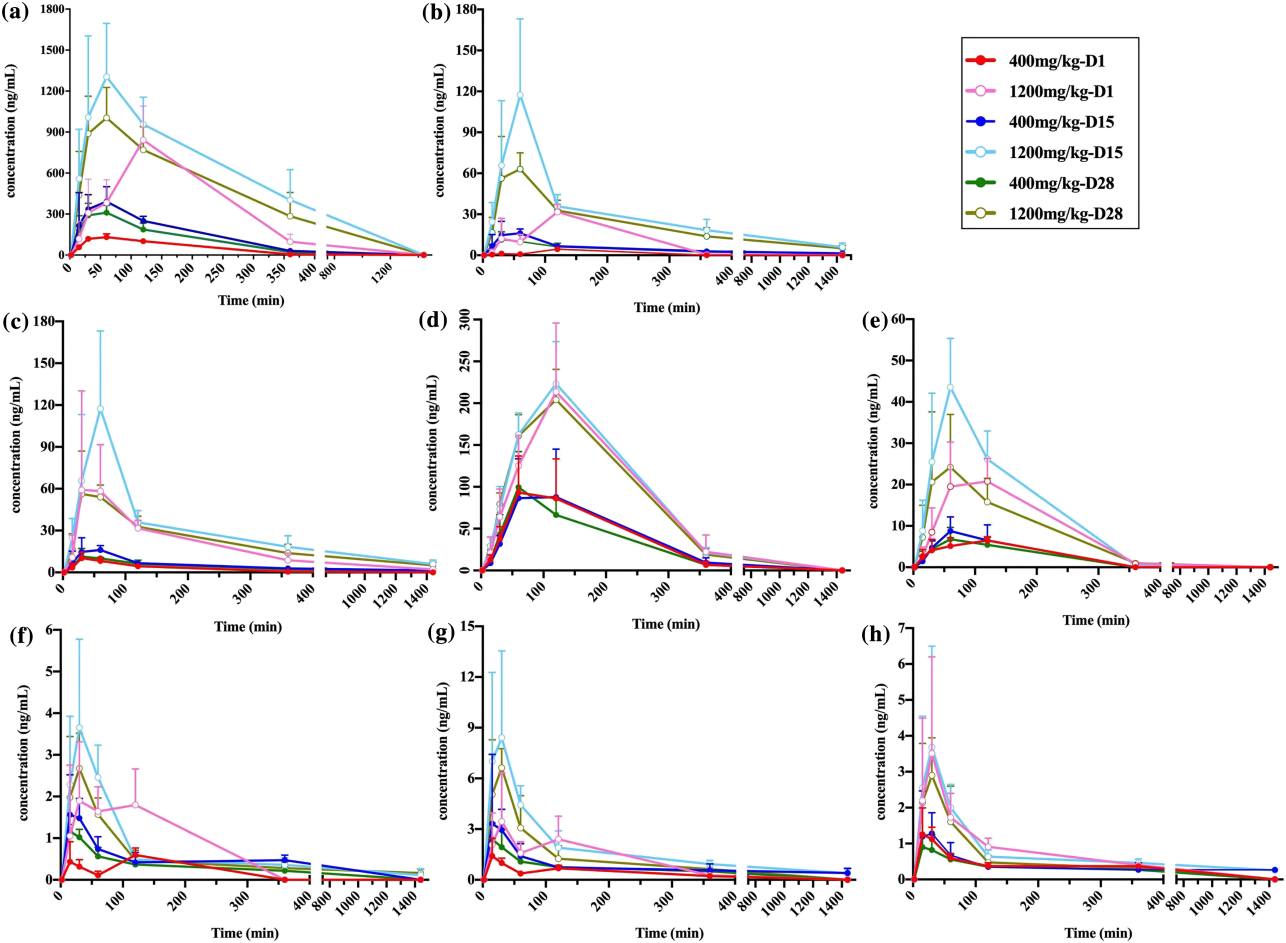
Mean plasma concentration–time profiles of (a) schisandrin, (b) schisantherin A, (c) schisandrin A, (d) prunasin, (e) amygdalin, (f) 3,4‐dihydroxybenzaldehyde, (g) genistein and (h) daidzein after administration of FZHY at doses of 400 and 1,200 mg/kg body weight, respectively, in beagle dogs

**FIGURE 4:**
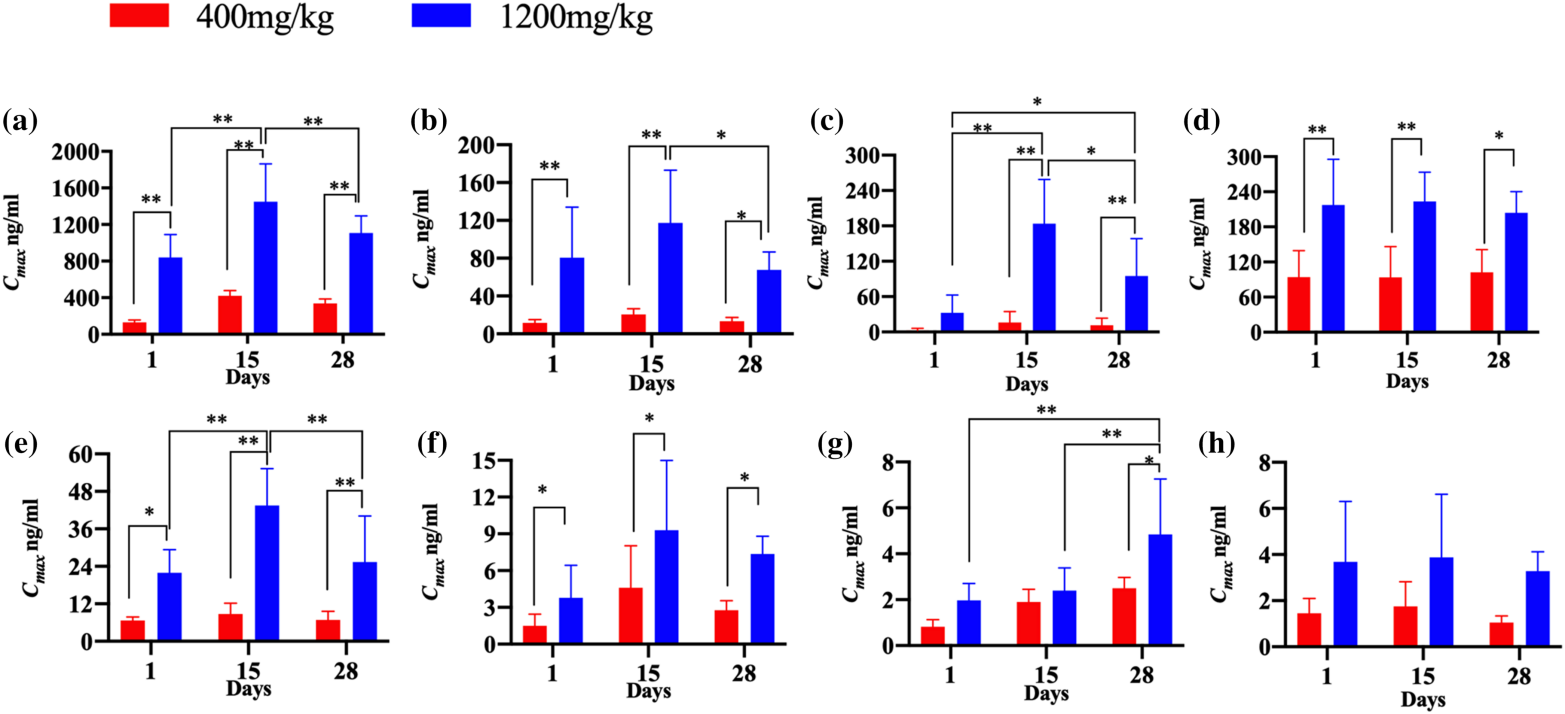
The *C*_max_ of (a) schisandrin, (b) schisandrin A, (c) schisantherin A, (d) prunasin, (e) amygdalin, (f) genistein, (g) 3,4‐dihydroxybenzaldehyde and (h) daidzein of beagle dogs after administration of FZHY at doses of 400 and 1,200 mg/kg (**P < 0.01 and *P < 0.05)

**FIGURE 5:**
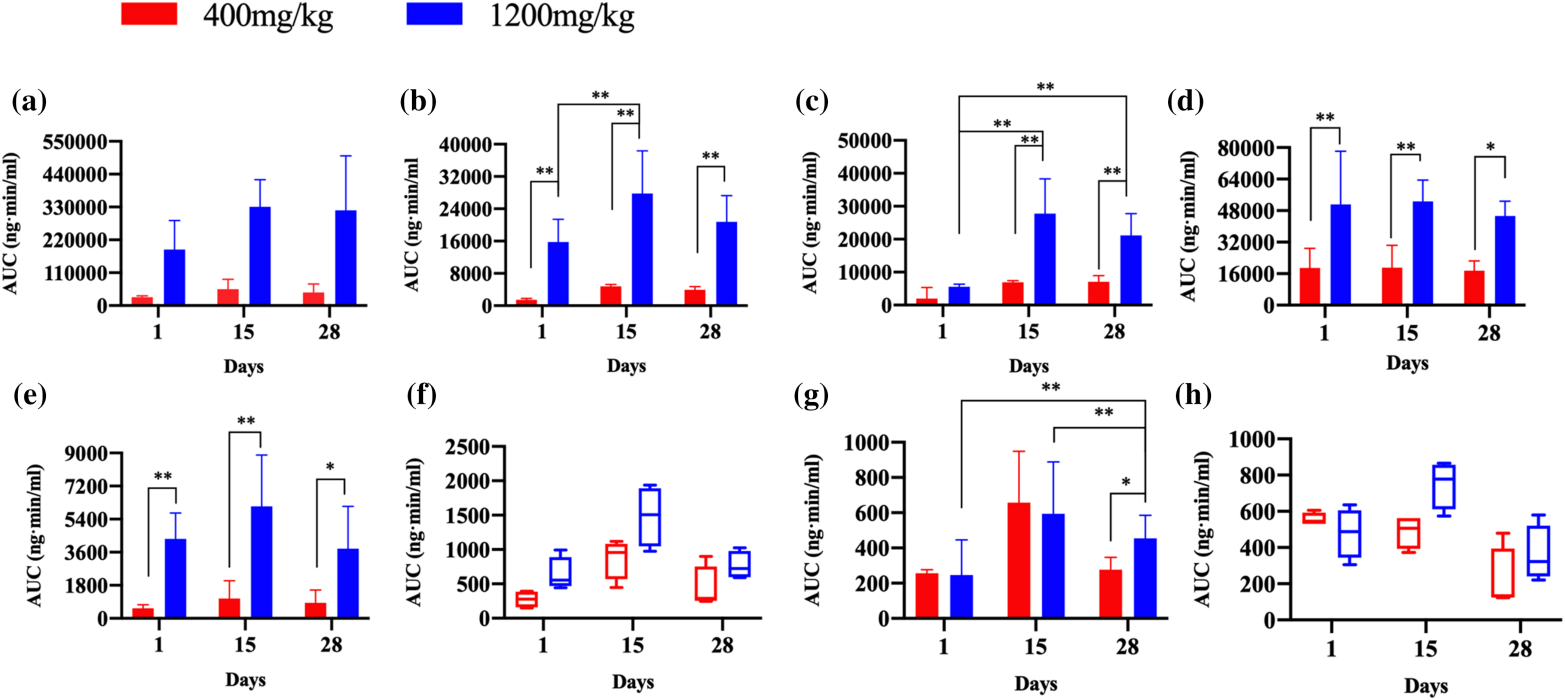
The AUC_(0–*t*)_ of (a) schisandrin, (b) schisandrin A, (c) schisantherin A, (d) prunasin, (e) amygdalin, (f) genistein, (g) 3,4‐dihydroxybenzaldehyde and (h) daidzein of beagle dogs after administration of FZHY at doses of 400 and 1,200 mg/kg (**P < 0.01 and *P < 0.05)

After single oral administration of FZHY at doses of 400 and 1,200 mg/kg for 1, 15 and 28 consecutive days, schisandrin, schisandrin A, schisantherin A, amygdalin, prunasin, genistein, 3,4‐dihydroxybenzaldehyde and daidzein were found at most sampling points using the UHPLC–Q‐Orbitrap HRMS method, and their toxicokinetic profiles and parameters were obtained (see Figures 3, 4, 5, and Table 3).

**TABLE 3:** Toxicokinetic parameters of the eight analytes after oral administration of Fuzheng Huayu recipe (FZHY) at a dose of 400 mg/kg and 1,200 mg/kg (mean ± SD, *M*[P25, P75], *n* = 4)

Analyte	Days	Dosage (ng/kg)	*C*_max_ (ng/ml)	*T*_max_ (min)	AUC_(0–t)_ (ng min/ml)	AUC_(0–∞)_ (ng min/ml)	*V*_d_ × 10^4^ (ml/kg)
Schisandrin	1	88.4	130.775 ± 24.97**	120.00 ± 0.00	28,116.35 ± 4,526.16	28,455.38 ± 4,567.28	0.23 ± 0.05
		265.2	841.50 ± 249.18^##^	120.00 ± 0.00	187,715.95 ± 96,885.17	193,548.88 ± 92,963.85	0.20 ± 0.00
	15	88.4	345.15 ± 198.92**	48.75 ± 22.5	54,896.18 ± 33,164.38	58,088.30 ± 35,253.00	1.33 ± 2.38
		265.2	1,450.73 ± 357.97^▲▲^	67.50 ± 32.69	330,388.65 ± 90,891.35	439,282.70 ± 172,258.47	0.18 ± 0.04
	28	88.4	264.55 ± 174.96*	45.00 ± 17.32	43,576.53 ± 28,560.84	45,801.88 ± 30,039.61	6.65 ± 12.97
		265.2	1,106.40 ± 188.87	45.00 ± 17.32	319,062.58 ± 182,616.95	339,949.10 ± 166,301.09	0.18 ± 0.05
Schisandrin A	1	93.3	11.55 ± 3.54**	45.00 ± 17.32	1,471.53 ± 323.38**	1,526.38 ± 319.55	10.18 ± 4.20
		280	80.48 ± 53.68^##^	60.00 ± 42.43	15,766.55 ± 5,667.43^##^	17,581.23 ± 6,832.61	13.40 ± 5.07
	15	93.3	20.60 ± 6.09**	52.50 ± 15.00	4,775.50 ± 468.24**	6,975.30 ± 539.93	21.30 ± 4.10
		280	117.48 ± 55.56^▲^	60.00 ± 0.00	27,793.43 ± 10,549.77	33,524.05 ± 13,465.70	8.73 ± 2.91
	28	93.3	13.35 ± 4.00*	45.00 ± 17.32	3,919.68 ± 847.92**	6,668.13 ± 1,636.34	29.65 ± 11.12
		280	67.53 ± 19.13	37.50 ± 15.00	20,752.50 ± 6,492.54	26,247.73 ± 8,662.26	12.58 ± 4.00
Schisantherin A	1	51.9	7.35 ± 7.18	75.00 ± 30.00	1,334.70 ± 2,233.10	1,887.18 ± 3,442.27	131.23 ± 277.47
		155.8	33.63 ± 3.07^##^	97.50 ± 45.00	5,535.80 ± 795.09^##^	5,566.83 ± 836.69	1.48 ± 0.13
	15	51.9	18.78 ± 7.94**	52.50 ± 15.00	4,792.48 ± 465.03**	6,916.38 ± 542.28	11.63 ± 2.31
		155.8	117.48 ± 55.56^▲^	60.00 ± 0.00	27,793.43 ± 10,549.77	33,524.05 ± 13,465.70	4.85 ± 1.63
	28	51.9	14.23 ± 4.40**	40.00 ± 17.32	3,642.30 ± 785.38**	6,620.23 ± 2,000.66	18.23 ± 6.32
		155.8	72.60 ± 18.25^△^	45.00 ± 17.32	21,163.98 ± 6,621.49^△△^	26,659.18 ± 8,793.13	6.90 ± 2.19
Amygdalin	1	269.1	6.65 ± 1.15*	97.50 ± 45.00	554.08 ± 193.81**	905.10 ± 3,388.21	76.30 ± 38.89
		807.4	21.95 ± 7.41^##^	90.00 ± 34.64	4,329.35 ± 1,406.99	4,397.30 ± 1,397.54	15.80 ± 7.18
	15	269.1	8.73 ± 3.43**	52.50 ± 15.00	1,078.70 ± 969.63**	1,696.25 ± 814.81	25.73 ± 14.20
		807.4	43.50 ± 11.82^▲▲^	60.00 ± 0.00	6,098.25 ± 2,788.65	6,787.10 ± 1,586.9	11.15 ± 9.61
	28	269.1	6.85 ± 2.72**	75.00 ± 30.00	849.90 ± 701.08*	817.90 ± 1,859.19	40.23 ± 8.58
		807.4	25.43 ± 14.72	45.00 ± 17.32	3,791.10 ± 2,311.95	4,332.30 ± 1,751.04	23.80 ± 15.36
3,4‐Dihydroxybenzaldehyde	1	103.8	1.43 ± 0.31	22.50 ± 48.02	256.23 ± 20.08	1,162.18 ± 118.86	299.50 ± 650.15
		311.3	2.73 ± 1.50	56.25 ± 46.44	245.78 ± 199.90	319.00 ± 289.96	241.38 ± 208.85
	15	103.8	3.78 ± 0.56	22.50 ± 7.5	656.73 ± 292.56	9,678.38 ± 684.84	104.20 ± 152.17
		311.3	5.80 ± 6.25^▲▲^	37.50 ± 15.00	594.68 ± 294.61^▲▲^	503.75 ± 665.52	1,087.18 ± 1,703.51
	28	29.1	2.53 ± 0.00*	30.00 ± 8.66	276.03 ± 71.58*	730.63 ± 680.41	34.70 ± 391.24
		311.3	3.85 ± 2.95^△△^	33.75 ± 18.87	455.15 ± 130.85^△△^	312.05 ± 789.97	983.70 ± 991.81
Genistein	1	29.1	1.50 ± 0.95*	18.75 ± 7.5	278.35 (160.78, 386.48)	1,180.58 ± 1,095.56	85.55 ± 48.17
		87.4	3.78 ± 2.65	71.25 ± 56.62		837.15 ± 382.55	70.95 ± 110.23
	15	29.1	4.60 ± 3.42*	26.25 ± 7.5	956.55 (569.70, 1,082.25)	10,080.38 ± 11,691.90	38.18 ± 9.88
		87.4	9.30 ± 5.68	48.75 ± 48.02		2,957.30 ± 1,746.38	61.03 ± 40.71
	28	29.1	2.78 ± 0.77*	30.00 ± 21.21	290.40 (254.88, 751.92)	1,573.00 ± 1,699.18	38.63 ± 10.52
		87.4	7.35 ± 1.46	26.25 ± 7.50		1,401.53 ± 908.21	58.55 ± 45.34
Daidzein	1	22.7	1.45 ± 0.65	22.50 ± 8.66	544.70 (531.30, 592.98)	11,129.65 ± 12,832.07	59.53 ± 3.84
		67.7	3.68 ± 2.62	41.25 ± 22.50		1,118.23 ± 1,167.69	65.20 ± 58.16
	15	22.7	1.75 ± 1.07	26.25 ± 7.50	505.95 (392.55, 561.83)	1,837.10 ± 2,698.46	75.40 ± 39.31
		67.7	3.88 ± 2.74	41.25 ± 22.50		1,507.05 ± 660.08	115.05 ± 57.03
	28	22.7	1.05 ± 0.29	30.00 ± 21.21	132.45 (123.75, 394.20)	776.53 ± 661.81	61.65 ± 8.00
		67.7	3.28 ± 0.84	33.75 ± 22.50		1,511.175 ± 4,398.25	127.43 ± 79.22
Prunasin	1	10.8	94.08 ± 45.20**	75.00 ± 30.00	18,959.55 ± 9,872.76**	19,590.08 ± 10,215.37	0.05 ± 0.06
		32.5	217.55 ± 77.98	105.00 ± 30.00	51,131.90 ± 26,993.09	51,693.40 ± 26,436.03	0.10 ± 0.00
	15	10.8	93.78 ± 52.37**	90.00 ± 34.64	19,013.73 ± 11,375.79**	20,034.55 ± 11,910.92	0.08 ± 0.05
		32.5	223.43 ± 50.17	120.00 ± 0.00	52,656.90 ± 10,784.26	53,460.78 ± 10,400.22	0.13 ± 0.05
	28	10.8	102.25 ± 38.94*	75.00 ± 30.00	17,526.35 ± 4,913.51*	18,056.18 ± 5,031.26	0.10 ± 0.08
		32.5	204.08 ± 36.31	120.00 ± 0.00	45,281.83 ± 7,536.42	46,701.93 ± 7,470.42	0.13 ± 0.05

*Notes*: ^**^ *P* < 0.01 and ^*^ *P* < 0.05, 400 mg/kg vs. 1,200 mg/kg; ^▲▲^ *P* < 0.01 and ^▲^ *P* < 0.05, 15 days vs. 28 days; ^##^ *P* < 0.01 and ^#^ *P* < 0.05, 1 day vs. 15 days; ^△△^ *P* < 0.01 and ^△^ *P* < 0.05, 1 day vs. 28 days.

#### 3.2.1 Toxicokinetic parameters of schisandrin, schisandrin A and schisantherin a

As shown in Figures 3, 4, 5 and Table 3, the toxicokinetic parameters of schisandrin, schisandrin A, and schisantherin A were similar, and the plasma concentration–time curve showed that these compounds were rapidly absorbed into the blood, reaching a maximum concentration, followed by a rapid decrease in concentration. Notably, the *C*_max_ values of schisandrin, schisandrin A and schisantherin A were all different after oral administration of FZHY at dosages of 400 and 1,200 mg/kg on days 1, 15 and 28, with significant differences observed (**P < 0.01, *P < 0.05). At a dosage of 400 mg/kg, the AUC_(0*–t*)_ values of schisandrin on days 1, 15 and 28 were 28,116.35 ± 4,526.16, 54,896.18 ± 33,164.38 and 43,576.53 ± 28,560.84 ng min/ml, respectively. When the dose was increased to 1,200 mg/kg, the concentration and the area under the drug–time curve became significantly larger. After administration for 15 days at 1,200 mg/kg, the largest AUC_(0*–t*)_ area (27,793.43 ± 10,549.77 ng·min/ml) was observed for schisandrin A. The AUC_(0*–t*)_ values of schisantherin A were 1,334.70 ± 2,233.10, 4,792.48 ± 465.03, and 3,642.30 ± 785.38 ng min/ml at 400 mg/kg and 5,535.80 ± 795.09, 27,793.43 ± 10,549.77 and 21,163.98 ± 6,621.49 ng·min/ml at 1,200 mg/kg, on days 1, 15 and 28, respectively (**P < 0.01, *P < 0.05). No significant differences were observed in the *T*_max_ and *V*_d_ values of schisandrin at high and low dosages on days 1, 15 and 28.

#### 3.2.2 Toxicokinetic parameters of amygdalin and prunasin

At FZHY doses of 400 and 1,200 mg/kg, the equivalent amygdalin doses administered were 269.1 and 807.4 ng/kg, while the prunasin content was much lower, at 10.8 and 32.5 ng/kg, respectively. Prunasin is a metabolite of amygdalin (Deng et al., 2021). The *C*_max_ values of amygdalin after FZHY administration at doses of 400 mg/kg (6.65 ± 1.15, 8.73 ± 3.43, and 6.85 ± 2.72 ng/ml) and 1,200 mg/kg (21.95 ± 7.41, 43.50 ± 11.82, and 25.43 ± 14.72 ng/ml) were much lower than those of prunasin at doses of 400 mg/kg (94.08 ± 45.20, 93.78 ± 52.37 and 102.25 ± 38.94 ng/ml) and 1,200 mg/kg (217.55 ± 77.98, 223.43 ± 50.17 and 204.08 ± 36.31 ng/ml), on days 1, 15 and 28, respectively. The AUC_(0*–t*)_ values of amygdalin were 6.65 ± 1.15, 8.73 ± 3.43, and 6.85 ± 2.72 ng min/ml at a dosage of 400 mg/kg, and 21.95 ± 7.41, 43.50 ± 11.82 and 25.43 ± 14.72 ng min/ml at a dosage of 1,200 mg/kg, which were significantly lower than those of prunasin.

#### 3.2.3 Toxicokinetic parameters of genistein, 3,4‐dihydroxybenzaldehyde, and daidzein

The toxicokinetic parameters of genistein, 3,4‐dihydroxybenzaldehyde and daidzein showed little or no significant differences among different dosages and days. The *C*_max_ values of genistein were different for FZHY doses of 400 and 1,200 mg/kg at each time point, namely, days 1 (1.50 ± 0.95 and 3.78 ± 2.65 ng/ml), 15 (4.60 ± 3.42 and 9.30 ± 5.68 ng/ml) and 28 (2.78 ± 0.77 and 7.35 ± 1.46 ng/ml) (**P < 0.01), and no significant difference in AUC_(0*–t*)_ values was observed among the different groups. The *C*_max_ values of 3,4‐dihydroxybenzaldehyde were 1.43 ± 0.31, 2.73 ± 1.50 ng/ml on day 1, 3.78 ± 0.56 and 5.80 ± 6.25 ng/ml on day 15 and 2.53 ± 0.00 and 3.85 ± 2.95 ng/ml on day 28 at a dosage of 1,200 mg/kg. A significant difference was observed on days 1, 15 and 28 (**P < 0.01, *P < 0.05). The AUC_(0–*t*)_ values of 3,4‐dihydroxybenzaldehyde were 256.23 ± 20.08, 656.73 ± 292.56, and 276.03 ± 71.58 ng min/ml at a dosage of 400 mg/kg, and slightly lower than those at a dosage of 1,200 mg/kg (245.78 ± 199.90, 594.68 ± 294.61 and 455.15 ± 130.85 ng min/ml) on days 1, 15 and 28, respectively (**P < 0.01, *P < 0.05). Unlike the two previous components, no significant differences were observed in the *C*_max_ and AUC_(0*–t*)_ values for daidzein.

## 4 DISCUSSION

By comparing different chromatographic columns and mobile phases, and adjusting the elution procedure, the Acquity HSS T3 column (2.1 × 100 mm, 1.8 μm) with gradient elution using 0.1% formic acid–water and acetonitrile solution was found to account for the physical and chemical properties of most analytes, and successfully separated the eight components (schisandrin, schisandrin A, schisantherin A, amygdalin, prunasin, genistein, 3,4‐dihydroxybenzaldehyde and daidzein).

An important step in drug toxicokinetic experiments is the choice of animal dose, which is directly related to the reliability of drug safety evaluation (Welling, 1995). The clinical dosage of FZHY tablets is 4.8 g/day (0.8 g/tablet × 2 tablets × 3 times per day). According to the conversion relationship between beagle dogs (10 kg) and humans (60 kg), the dosage for beagle dogs was 1.8 tablets/kg/day (144 mg/kg/day). The maximum tolerated dose test showed no abnormalities from a single administration of six tablets to beagle dogs, while symptoms of loose stool and vomiting were observed when 18 tablets (nine tablets, twice per day) or 45 tablets (15 tablets, three times per day) were administered within 24 h. The requirement for toxicokinetic studies is to set three dose groups of low, medium and high concentration. The low dose is a dose without a toxic reaction, the medium dose is a dose reflecting lower toxicity and a high dose should have obvious toxicity. As the purpose of the low dose is to provide a reference for the clinical dose, the final dosages were set at 400 and 1,200 mg/kg. The route of administration, commonly oral administration clinically, is chosen to accurately reflect the toxicokinetic behavior of beagle dogs after long‐term high‐dose administration. Toxicokinetic experiments require male and female animals and a certain number of animals. The blood sampling time point should reach the required frequency, but not be so frequent as to interfere with normal toxicological research. The subject of this study is the beagle dog, which has a large body with a small amount of blood taken. Each group comprised four animals, two female and two male. Based on the results of the preliminary experiment, and considering the drug peak and elimination, seven blood sampling points were set for regular blood collection to reflect systemic exposure of the drug. The blood volume taken from each animal was <10% of the circulating blood volume to ensure no interference with normal research of the animal and avoid causing excessive physiological stress.

Previous pharmacokinetic studies on FZHY have mainly focused on rats. The pharmacokinetics of six lignan components contained in FZHY, namely, schisandrin A, schisandrin, schisandrin C, schisandrol A, schisandrol B and schisantherin A, has been reported. The pharmacokinetic properties of danshensu, salvianolic acid B, rosmarinic acid and amygdalin in normal and fibrotic rats have also been studied (Yang, Liu, Wang, et al., 2015b; Yang, Liu, Zheng, et al., 2015). The pharmacokinetics of three lignans and amygdalin in rats has been studied. In contrast to previous studies, the present study focused on investigating the plasma concentration and toxicokinetics of chemical components of FZHY in beagle dogs. Pre‐experiments showed that, in addition to schisandrin, schisandrin A, schisantherin A and amygdalin, prunasin, genistein, 3,4‐dihydroxybenzaldehyde and daidzein all had a clear dynamic process in beagle dogs. Therefore, these eight components were finally selected for the toxicokinetic study. The pharmacokinetic parameters of the four components of FZHY – schisantherin A, schisandrin A, schisandrin and amygdalin – were compared between rats and beagle dogs at a dose equivalent to three times that supplied to humans. The *T*_max_ and AUC_(0–*t*)_ values also showed differences between rats and beagle dogs. The *T*_max_ values of schisantherin A, schisandrin A and schisandrin were higher in dogs, while the *T*_max_ value of amygdalin was lower in dogs. Furthermore, the AUC_(0–*t*)_ values of schisantherin A and schisandrin A in beagle dogs were significantly lower than those in rats. Altogether, these results indicated the difference between rat and beagle dog species.

According to the statistical results and the main toxicokinetic parameters of the eight analytes, the blood concentrations of 3,4‐dihydroxybenzaldehyde and daidzein quickly reached their peak (*T*_max_) at a low concentration level. This suggested that 3,4‐dihydroxybenzaldehyde and daidzein were quickly absorbed and eliminated, in accordance with previous results showing that the free and glycoside forms of genistin are readily bioavailable (Steensma et al., 2006). The AUC_(0*–t*)_ value of schisandrin was large, showing high systemic exposure. However, the AUC_(0*–t*)_ value did not increase with prolonged administration time at the same dose, and no accumulation in the body was observed. The AUC_(0*–t*)_, *T*_max_ and *C*_max_ of schisandrin have been reported to decrease after administration with *Fructus Schisandrae Chinensis* water extract for 3 weeks compared with one administration, which is similar to the phenomenon observed in the present study, and also demonstrates the safety of schisandrin for long‐term use (Gao et al., 2019). Analytes schisandrin A, schisandrin A, amygdalin and genistein also showed no significant accumulation in beagle dogs on days 1, 15 and 28 of FZHY administration. With extended administration time, the *in vivo* exposure became smaller, and the AUC_(0*–t*)_ value on day 28 was smaller than that on day 15.

No obvious difference in the AUC_(0*–t*)_ value of prunasin was observed with dosage and administration time, indicating no accumulation in the body at this dosage. Notably, amygdalin is nontoxic and has an anti‐liver fibrosis effect by decreasing the mRNA and protein expression levels of CTGF and TGF‐*β* (Luo et al., 2016), and inhibiting the expression of platelet‐derived growth factor and insulin‐like growth factor mRNA (Luo et al., 2018). Prunasin is a degradation product of amygdalin, which can be hydrolyzed to mandelonitrile and glucose by the action of enzyme prunasin lyase; mandelonitrile is then broken down into benzaldehyde by hydroxyl nitrile lyase, with the release of hydrogen cyanide, which is harmful to health (Go et al., 2018). Therefore, the AUC results showed that amygdalin was extensively transformed into prunasin *in vivo*, and that the efficacy and toxicity of prunasin should receive more attention.

The blood concentrations of genistein, 3,4‐dihydroxybenzaldehyde and daidzein were low at a dosage of 1,200 mg/kg, and there was no significant difference in the AUC_(0*–t*)_ values of genistein and daidzein at 1, 15 and 28 days, indicating that the *in vivo* exposure was low and stable without accumulation. Genistein and daidzein are important isoflavones, and several experimental and clinical investigations have shown that genistein can treat different types of cancer (Jin et al., 2009; Yan et al., 2005). However, the blood concentration of genistein in dog plasma was low after FZHY administration, with studies showing that potential toxicity and side effects will occur only when the genistein concentration used is much higher than the physiological dose available through diet or drug intake (Carmela et al., 2015). For daidzein, the low bioavailability was closely related to low solubility in water, the oil–water partition coefficient and intensive metabolism in the intestine and liver (Kwiecien et al., 2020). Owing to the small number of male and female animals used, no gender comparison was performed in this experiment, and tissue distribution will be investigated in the future.

## 5 CONCLUSION

The eight components of FZHY (schisandrin, schisandrin A, schisantherin A, amygdalin, prunasin, genistein, 3,4‐dihydroxybenzaldehyde and daidzein) were detected using a UHPLC–Q‐Orbitrap method, which is rapid, accurate and sensitive. Toxicokinetic parameters, including *C*_max_, *T*_max_, AUC_(0–*t*)_, AUC_(0*–∞*)_ and *V*_d_ values, were obtained for beagle dogs, with no obvious accumulation observed after administration of FZHY doses of 400 and 1,200 mg/kg/day on days 15 and 28 during the study.

## FUNDING STATEMENT

This work was supported by the Shanghai International Science and Technology Cooperation Project (grant number 18400731400).

## AUTHORS’ CONTRIBUTIONS

Tao Yang and Yu‐lin Wang conceived and designed the study. Tao Yang, Yu‐lin Wang, Ming‐jv Yang and Rong‐sheng Li performed most of the experiments. Ye‐qing Hu contributed to the experiments. Yi‐feng Pan provided resources. All authors discussed the results. Yu‐lin Wang and Ming‐jv Yang wrote the paper with input from all authors.

## CONFLICT OF INTEREST

The authors declare that there are no conflicts of interest.

## Supporting information


**Table S1** Calibration curve, *R* ^2^, Linear range, LLOQ and LOD of the eight analytes
**Table S2** Concentration of low, medium and high QC samples
