# Supplementary material for: Simultaneous determination of eight analytes of Fuzheng Huayu recipe in beagle dog plasma by UHPLC–Q/exactive Orbitrap HRMS and its application to toxicokinetics
Source: Biomed Chromatogr. 2022 Jan 22;36(4):e5329. doi: 10.1002/bmc.5329 (PMC9287080; doi:10.1002/bmc.5329)
Supplement: Supplementary file 1 — Table S1 Calibration curve, R 2, Linear range, LLOQ and LOD of the eight analytes Table S2 Concentration of low, medium and high QC samples [file BMC-36-0-s001.docx]

**Supplementary results**

**Table S1** Calibration curve, R^2^, Linear range, LLOQ, and LOD of the eight analytes

| Analyte | Calibration  curve | R^2^ | Linear range  (ng/ml) | LOD (ng/ml) | LLOQ (ng/ml） |
| --- | --- | --- | --- | --- | --- |
| schisandrin | y = 0.0014x + 0.0428 | 0.9875 | 1.32-800.00 | 0.21 | 1.32 |
| schisandrin A | y = 0.0061x + 0.1046 | 0.9879 | 0.21-51.20 | 0.08 | 0.21 |
| schisantherin A | y = 0.0044x + 0.0655 | 0.9914 | 0.21-51.20 | 0.08 | 0.21 |
| amygdalin | y = 0.0005x + 0.0062 | 0.9934 | 0.08-20.48 | 0.03 | 0.08 |
| prunasin | y = 0.0012x + 0.0145 | 0.9933 | 0.53-128.00 | 0.08 | 0.53 |
| genistein | y = 0.0049x + 0.1065 | 0.9851 | 0.08-8.19 | 0.03 | 0.08 |
| daidzein | y = 0.0046x + 0.1114 | 0.9820 | 0.08-8.19 | 0.03 | 0.08 |
| 3,4-dihydroxybenzaldehyde | y = 0.0041x + 0.129 | 0.9875 | 0.03-3.28 | 0.01 | 0.03 |

**Table S2** The concentration of low, medium, and high QC samples

| Analyte | LQC (ng/ml) | MQC (ng/ml) | HQC (ng/ml） |
| --- | --- | --- | --- |
| schisandrin | 3.28 | 51.20 | 800.00 |
| schisandrin A | 0.53 | 20.48 | 51.20 |
| schisantherin A | 0.53 | 8.19 | 51.20 |
| amygdalin | 0.21 | 3.28 | 20.48 |
| prunasin | 1.32 | 8.19 | 128.00 |
| genistein | 0.21 | 1.32 | 8.19 |
| daidzein | 0.21 | 1.32 | 8.19 |
| 3,4-dihydroxybenzaldehyde | 0.08 | 0.53 | 3.28 |
